# Association of antiretroviral therapy regimens with serum cortisol abnormalities in people living with HIV/AIDS: a retrospective study

**DOI:** 10.3389/fendo.2026.1841173

**Published:** 2026-06-26

**Authors:** Han Yajuan, Chen Xingxing, Jiang Huirong, Wan Jinshu, Zhang Yinhua, Pan Xiuzhen, Muhammad Tahir Khan, Xu Chao, Lin Sizhe, Pan Chih-Lin, Liu Yanrong, He Piao

**Affiliations:** 1 Department of Endocrinology, Guangzhou Eighth People’s Hospital, Guangzhou Medical University, Guangzhou, China; 2 International Center for Interdisciplinary Research in Sciences (ICIRS), The University of Lahore, Lahore, Pakistan; 3 State Key Laboratory of Respiratory Disease, Guangzhou Key Laboratory of Tuberculosis Research, Department of Clinical Laboratory, Guangzhou Chest Hospital, Institute of Tuberculosis, Guangzhou Medical University, Guangzhou, China

**Keywords:** abnormal blood cortisol levels, art, cholesterol, diagnosis, endocrine, HIV/AIDS

## Abstract

**Introduction:**

The life expectancy of people living with HIV (PLWH) has significantly increased, largely due to the antiretroviral therapy (ART) used very frequently.

**Objective:**

To explore the associated factors of abnormal serum cortisol levels in PLWH and the correlation between different ART regimens.

**Methods:**

A retrospective cross-sectional study was conducted using clinical data of people living with HIV between May 2017 and March 2025.

**Results:**

In this study involving 117 PLWH, 56 cases (47.9%) exhibited an abnormal high cortisol level. Subjects were stratified into morning serum hypocortisolemia group (Group A), morning serum normocortisolemia (Group B), and abnormal morning serum hypercortisolemia (Group C). Group A exhibited significantly higher diastolic blood pressure, longer duration of HIV diagnosis, and higher ART utilization rate. Additionally, the duration of HIV/AIDS Group A diagnosis was significantly longer and exhibited significant differences (*P* < 0.05) when compared with Group C. The mean triglyceride (TG) level in Group C was significantly elevated compared to that in Group B, and the low-density lipoprotein cholesterol (LDL-C) level in Group A was significantly higher than that in Group C (*P* < 0.05). The final logistic regression model was statistically significant (χ^2^ = 138.00, *P* = 0.008), and the parallel lines test (χ^2^ = 18.998, *P* = 0.123). The duration of HIV diagnosis was associated with changes in blood cortisol levels (OR = 0.987, 95% CI: 0.97–0.99, P = 0.042). In phase II, among 73 PLWH individuals receiving stable ART regimens, the proportion of decreased cortisol differed significantly among ART regimen groups (P < 0.05). Binary logistic regression analysis showed that longer duration of HIV diagnosis (OR = 1.02, P = 0.021) and the regimen (nucleoside reverse transcriptase inhibitors plus protease inhibitors (NRTIs + PIs) (OR = 5.36, P = 0.034)) were independently associated with reduced cortisol levels.

**Conclusion:**

The NRTIs + PIs regimen was associated with a significantly higher likelihood of reduced cortisol compared with the NRTIs + NNRTIs regimen. This finding was supported by multivariate logistic regression, which indicated that regimen (2) (NRTIs + PIs) was an independent factor associated with decreased cortisol levels.

## 1 Introduction

Acquired Immunodeficiency Syndrome (AIDS) remains one of the major public health issues caused by HIV (Human Immunodeficiency Virus) (1, 2). The Joint United Nations Program on HIV/AIDS (UNAIDS) estimated that 39.9 million people were living with HIV/AIDS by the end of 2023, with about 1.3 million newly diagnosed cases and 0.6 million deaths (3). The prevalence of HIV infections varies significantly across Sub-Saharan Africa, where it has the highest rates, with prevalence exceeding 10% in many countries (4, 5). Infection rates in Southeast Asia and Eastern Europe are relatively lower, but an increasing trend has been observed in recent years (4, 6). With the implementation of public health interventions, the incidence of AIDS has declined in some countries; however, overall trends are still influenced by socioeconomic factors, healthcare resource allocation, and cultural practices (7, 8).

In China, since the first case of HIV infection was reported in 1985, the incidence of has increased yearly, particularly among young men. The incidence in males aged 20–35 years is significantly higher than that in females (9). Studies indicate that in 2024, the number of newly reported HIV/AIDS cases in China reached 101,600, suggesting active transmission of the disease among the population (10). In addition, significant regional differences in HIV incidence have been reported within China, with eastern coastal regions generally lower than western and inland areas, which is closely related to higher levels of economic development and greater availability of medical resources in the former regions (11).

HIV is a retrovirus that is mainly classified into HIV-1 and HIV-2. HIV-1 is the predominant type worldwide, whereas HIV-2 is primarily prevalent in West Africa (12). Structurally, HIV consists of an outer lipid bilayer membrane and an inner spherical viral core composed of two positive-sense RNA and multiple viral proteins (13, 14). The viral envelope contains two major glycoproteins, gp120 and gp41. gp120 is responsible for binding to the CD4 receptor on the surface of host cells, while gp41 mediates fusion between the viral and host cell membranes. These structural features enable HIV to infect CD4^+^ T lymphocytes efficiently. With continuous viral replication, the immune system is progressively damaged, CD4^+^ T-cell counts gradually decline, and eventually the immune system collapses (15).

With the widespread use of ART, the survival of people living with HIV (PLWH) has been markedly prolonged, and many PLWH can now live near-normal lives. Some studies have reported that the five-year survival rate of PLWH receiving ART can reach 72%–84% (16, 17). Moreover, early initiation of ART is closely associated with better survival outcomes, particularly in PLWH with low CD4^+^ T-cell counts (18, 19). However, while ART has significantly reduced AIDS-related mortality, ART-associated metabolic abnormalities have become increasingly common, including disorders of glucose and lipid metabolism, thyroid dysfunction, adrenal cortical dysfunction, and hypogonadism (20, 21).

In recent years, abnormal cortisol levels have become one of the most common endocrine abnormalities in PLWH. Abnormal cortisol levels are closely associated with the occurrence and progression of various diseases, such as metabolic syndrome, Addison’s disease, depression, and cardiovascular diseases (18, 22, 23). Consequently, increasing attention has been paid to the impact of ART on cortisol levels in PLWH (24). Cortisol is a glucocorticoid hormone secreted by the zona fasciculata of the adrenal cortex (25). Circadian rhythms and stress conditions regulate its secretion, and it participates in the regulation of multiple physiological processes, including metabolism, immune responses, and stress responses.

This study aims to explore the factors associated with abnormal serum cortisol levels in PLWH and the correlation between ART regimens. First, understanding the factors underlying cortisol abnormalities may help clinicians to intervene early in patient management. Second, by analyzing the association between ART and reduced blood cortisol levels, this study may inform clinical practice and help clinicians develop individualized treatment strategies. This study will also lay a foundation for future research and promote deeper exploration of endocrine and metabolic abnormalities in PLWH. The findings will provide evidence for the formulation of public health policies, facilitate comprehensive management of PLWH, and improve their quality of life and life expectancy. Through these efforts, we hope to contribute to improving the overall health status of individuals living with HIV/AIDS.

## 2 Methods and materials

The study was performed in two phases. Phase I included all eligible people living with HIV (PLWH) who underwent their first serum cortisol measurement during the study period, and participants were categorized by serum cortisol level (reduced, normal, or elevated). This phase focused on the overall distribution and clinical characteristics associated with cortisol status.

Phase II is a subgroup analysis derived from Phase I. Only PLWH receiving antiretroviral therapy (ART) with relatively stable regimens were included to evaluate the association between different ART regimens and cortisol levels. These criteria were introduced to minimize potential confounding effects from treatment instability and to improve comparability between ART regimen groups.

The ART utilization rate refers to the proportion of people living with HIV (PLWH) who were receiving ART at the time of cortisol assessment among the total number of participants in each study group.

Participants were stratified by ART regimen at the time of sample collection. The patients were categorized into predefined ART groups for comparative analyses. Regimen classification was performed according to the major antiretroviral drug.

### 2.1 Phase I: study subjects

#### 2.1.1 Ethical approval and study subjects

Ethical approval was taken from the Ethics Committee of the Guangzhou Medical University Affiliated Eighth People’s Hospital((2020) 33166). Informed consent was waived because this study involved a retrospective analysis of anonymized data and posed no risk to participants. All data were fully ensuring participant confidentiality in accordance with applicable ethical guidelines and the Declaration of Helsinki.

PLWH who attended the Infectious Diseases Unit at the Eighth People’s Hospital of Guangzhou Medical University between May 2017 and March 2025 and underwent blood cortisol testing were enrolled.

A total of 117 PLWH met the inclusion criteria, including 83 males (70.9%) and 34 females (29.1%). The median duration of HIV diagnosis (defined as the time from HIV diagnosis to blood cortisol testing) was 43.00 months.

Blood cortisol levels of 133.0–537.0 nmol/L were defined as normal; values below this range were considered decreased, and values above this range were considered elevated. Based on these criteria, the enrolled PLWH were divided into three groups: Group A (decreased blood cortisol), 44 cases (37.6%); Group B (normal blood cortisol), 61 cases (52.1%); and Group C (elevated blood cortisol), 12 cases (10.3%). Differences among the three groups were compared to analyze factors associated with abnormal cortisol levels in blood. A retrospective study design was employed, and relevant data, including medical records, medical orders, and laboratory and examination results, were retrieved for analysis.

### 2.2 Study methods

#### 2.2.1 Phase I:

##### 2.2.1.1 Inclusion criteria

The inclusion criteria include (1); Confirmed HIV infection, meeting the diagnostic criteria for HIV infection by any one of the following: (1) +HIV antibody test and HIV confirmatory test (+ antibody confirmatory test, or + nucleic acid, or quantitative nucleic acid >1,000 copies/mL); (2) Presence of epidemiological history or AIDS-related clinical manifestations with two positive HIV nucleic acid tests; (3) Positive HIV isolation test.

**Figure 1:**
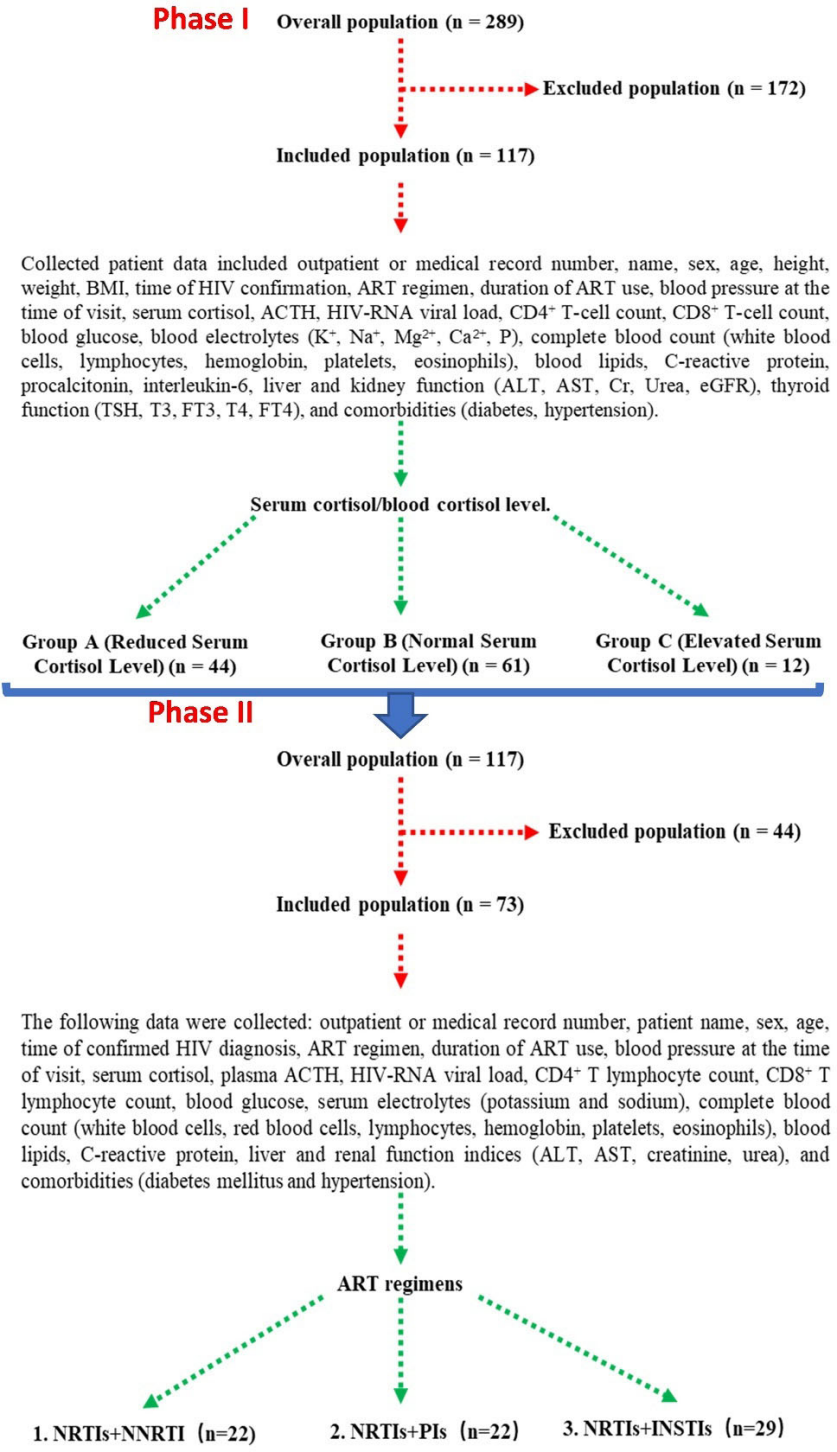
Phase II flowchart methodology of sample collection and cortisol level.

(2) PLWH who had blood drawn for cortisol testing for the first time between May 2017 and March 2025, with sampling performed between 6:00 and 10:00 a.m (See **Figure 1**, Phase 1).

##### 2.2.1.2 Exclusion criteria

(1) PLWH with a confirmed diagnosis of Cushing’s syndrome, primary hyperaldosteronism, pheochromocytoma, small-cell lung cancer, adrenocortical carcinoma, adrenal metastatic carcinoma, congenital adrenal hyperplasia, ganglioneuroma, paraganglioma, schwannoma, adrenal hemorrhage; those with concomitant infections, concomitant tuberculosis, or diabetic ketoacidosis within the past 3 months (2); Pregnant or lactating PLWH (3); PLWH under 18 years of age;

(4) PLWH who had used oral or intravenous glucocorticoids, ketoconazole, phenytoin sodium, estrogen, rifampicin, or other drugs affecting hypothalamic–pituitary–adrenal (HPA) axis rhythms within the past 3 months;

(5) PLWH with severely incomplete medical records.

### 2.3 Clinical data

The patient data were retrieved from the electronic medical record for individuals who attended the Department of Infectious Diseases and underwent blood cortisol testing. PLWH data were analyzed according to the inclusion and exclusion criteria. From the eligible cases, the following clinical data were collected:

### 2.4 General patient information

inpatient or outpatient number, name, sex, age, weight (kg), height (cm), body mass index (BMI, kg/m²), systolic and diastolic blood pressure (mmHg) at the time of visit, time of HIV confirmation, past medical history, current ART regimen, recent medication use, and comorbidities such as diabetes mellitus and hypertension.

All enrolled PLWH had blood samples collected (between 6:00 and 10:00 a.m) for cortisol measurement. Concurrent laboratory data (with all tests completed within 1 month) were also collected, including adrenocorticotropic hormone (ACTH), HIV-RNA virus load, T-lymphocyte count-CD4^+^, T-lymphocyte count-CD8^+^, triglycerides (TG), total cholesterol level (TC), high density lipoprotein-cholesterol (HDL-C), low density lipoprotein-cholesterol (LDL-C), alanine aminotransferase (ALT), total bilirubin (TB), serum creatinine (Cr), aspartate aminotransferase (AST), blood urea (Urea), direct bilirubin (DB), estimated glomerular filtration rate (eGFR), random blood glucose (Glu), white blood cell count (WBC), lymphocyte percentage (Lymp%), absolute lymphocyte count (Lymph), eosinophil percentage (Eo%), eosinophil count (Eo), red blood cell count (RBC), platelet count (PLT), hemoglobin (HGB), procalcitonin (PCT), potassium (K^+^), sodium (Na^+^), calcium (Ca²^+^), phosphorus, magnesium (Mg²^+^), thyroid-stimulating hormone (TSH), free thyroxine (FT4), thyroxine (T4), free triiodothyronine (FT3), and triiodothyronine (T3). Quantitative measurement of blood cortisol was outsourced to Guangzhou KingMed Diagnostics and performed using a Siemens electrochemiluminescence immunoassay.

### 2.5 Data analysis and processing

Statistical analyses and correlation analyses were performed using SPSS version 25.0. Normally distributed continuous variables were described as mean ± standard deviation ( $\bar{x}$ ± SD), whereas non-normally distributed continuous variables were described as median (interquartile range) [M (P25, P75)]. Categorical variables were expressed as rates or proportions (%).

For comparisons of continuous variables among multiple groups, one-way analysis of variance (ANOVA) with Bonferroni *post hoc* multiple comparisons was used for normally distributed data. For non-normally distributed data, the Kruskal–Wallis rank-sum test was applied. Categorical data were analyzed using the χ² test; when the total sample size or the minimum expected frequency did not meet the requirements for the χ² test, Fisher’s exact probability test was used. For multiple comparisons in chi-square tests, the α-splitting method was applied for pairwise comparisons, and an adjusted P value (adj. P) < 0.05 was considered statistically significant.

Blood cortisol status (decreased, normal, or elevated) was the dependent variable, and other variables were included as independent variables in an ordinal multivariate logistic regression model to estimate odds ratios (ORs) and 95% confidence intervals (95% CIs).

In the ordinal logistic regression analysis, cortisol status (decreased, normal, or elevated) was the dependent variable. Variables showing significant differences in the univariate analyses were included as independent variables in the multivariable model. Variables with more than 20% missing observations were excluded from statistical analyses before model construction, and the remaining analyses were performed using available data. Odds ratios (ORs) and 95% confidence intervals (95% CIs) were calculated. All statistical tests were two-sided, and P < 0.05 was considered statistically significant.

### 2.6 Phase II: analysis of the association between different antiretroviral therapy regimens and decreased blood cortisol levels in PLWH

#### 2.6.1 Study subjects

PLWH who attended the Department of Infectious Diseases at the Eighth People’s Hospital of Guangzhou Medical University between May 2017 and March 2025 were selected. Eligible participants were determined according to the inclusion and exclusion criteria. A total of 73 PLWH were ultimately included. PLWH with elevated serum cortisol (n = 6) and those receiving ART regimens in which all three antiretroviral drugs were nucleoside reverse transcriptase inhibitors (NRTIs) only (n = 1) were excluded from this part of the analysis due to the extremely small sample size.

Among the included PLWH, 49 were males (67.1%) and 24 were females (32.9%). Thirty-five PLWH (47.9%) had normal serum cortisol levels, and 38 PLWH (52.1%) had decreased serum cortisol levels. The median duration since HIV diagnosis was 56.50 months. Serum cortisol levels between 133.0 and 537.0 nmol/L were defined as normal, while values below this range were defined as decreased.

Based on the class of the third drug in the current ART regimen, the study population was divided into three groups to examine the association between ART regimens and decreased serum cortisol levels. A retrospective study design was adopted, and relevant data were extracted from PLWH’ medical records, physician orders, and laboratory test results.

#### 2.6.2 Study methods

##### 2.6.2.1 Study inclusion criteria

(1) Confirmed HIV infection, requirement for HIV diagnostic criteria, defined as fulfillment of any one of the following:

- Positive HIV antibody and confirmatory tests (+ive antibody confirmatory test, or +ive qualitative nucleic acid test, or quantitative nucleic acid test >1,000 copies/mL);
- Presence of epidemiological exposure history or AIDS-related clinical manifestations, with two consecutive positive HIV nucleic acid tests;
- Positive HIV isolation test.

(2) PLWH who underwent their first serum cortisol measurement between 6:00 and 10:00 a.m. during the study period from May 2017 to March 2025.

(3) PLWH receiving ART who had not changed their ART regimen, or who had changed regimens but had been on the current regimen for at least 90 days.

##### 2.6.2.2 Exclusion criteria

1. PLWH with a confirmed diagnosis of Cushing’s syndrome, primary aldosteronism, pheochromocytoma, small cell lung cancer, adrenocortical carcinoma, adrenal metastatic carcinoma, congenital adrenal hyperplasia, ganglioneuroma, paraganglioma, schwannoma, adrenal hemorrhage, concomitant infections, tuberculosis, or diabetic ketoacidosis within the past three months;
2. Pregnant or lactating individuals;
3. Individuals under 18 years of age;
4. PLWH who had received oral or intravenous glucocorticoids, or drugs affecting HPA axis rhythm such as ketoconazole, phenytoin sodium, estrogen, or rifampicin within the previous three months (5); PLWH with severely incomplete medical records (See **Figure 1**, Phase II).

### 2.7 Clinical data

In this study, the hospital’s digital medical record system was used to retrieve data for PLWH hospitalised in the Infectious Diseases Department or attending outpatient visits who underwent blood cortisol testing. PLWH were screened according to the inclusion and exclusion criteria, and the following clinical data were recorded from the selected cases:

General patient information: Hospital or outpatient number, name, age, sex, body weight (kg), blood pressure at the time of visit (systolic and diastolic, mmHg), duration since confirmed HIV diagnosis, past medical history, current ART regimen, recent medication use, and comorbidities such as diabetes and hypertension.

All enrolled PLWH had blood samples collected (between 6:00 and 10:00 a.m.) for cortisol measurement. Concurrently (all tests completed within one month), the following laboratory data were collected: adrenocorticotropic hormone (ACTH), HIV-RNA viral load, CD4+ T lymphocyte count, CD8+ T lymphocyte count, triglycerides (TG), total cholesterol (TC), low-density lipoprotein cholesterol (LDL-C), high-density lipoprotein cholesterol (HDL-C), aspartate aminotransferase (AST), alanine aminotransferase (ALT), direct bilirubin (DB), total bilirubin (TB), serum creatinine (Cr), random blood glucose (Glu), blood urea (Urea), lymphocyte percentage (Lymp%), white blood cells (WBC), absolute lymphocyte count (Lymph), eosinophil percentage (Eo%), eosinophil count (Eo), red blood cells (RBC), hemoglobin (HGB), platelets (PLT), potassium (K+), and sodium (Na+).

Quantitative measurement of blood cortisol was performed externally at Guangzhou KingMed Medical Laboratory using the Siemens electrochemiluminescence method.

### 2.8 Data analysis and processing

Data were analyzed using SPSS 25.0 statistical software. Continuous variables with normal distribution were expressed as mean ± standard deviation ( $\bar{X}$ ± S\bar{X} \pm S $\bar{X}$ ± S), while non-normally distributed continuous variables were expressed as median (interquartile range) [M (P25, P75)]. Categorical variables were expressed as rates or percentages (%).

For comparisons among multiple groups:

• Continuous variables with normal distribution were analyzed using one-way ANOVA (with Bonferroni multiple comparison correction).
• The Kruskal-Wallis test was applied for non-normally distributed variables.
• The Chi-square test (χ2\chi^2χ2) was used for categorical variables if the total counts or minimum expected frequency did not meet Chi-square requirements; Fisher’s exact test was used.

For multiple chi-square tests, the α-splitting method was used for pairwise comparisons. P values (adj.P) < 0.05 were considered statistically significant for the analysis of differences.

Binary Logistic regression analysis with forward: LR method was performed, using cortisol reduction (yes/no) as the dependent variable and other variables as independent variables to calculate OR (odds ratios) and 95% CI (95% confidence intervals). All statistical tests were two-sided, and a statistically significant result was considered at P < 0.05.

## 3 Phase I results

### 3.1 Clinical data and laboratory results

The median age of the study population was 40.00 years (interquartile range: 32.00–54.50). The median blood cortisol level was 258.00 nmol/L (21.75–431.40). Among the participants, 44 cases (37.6%) had decreased cortisol, 61 cases (52.1%) had normal cortisol, and 12 cases (10.2%) had elevated cortisol.

98 PLWH (83.8%) were receiving ART, while 19 PLWH (16.2%) were not. The median ACTH level was 6.33 pmol/L (2.98–12.48). The median CD4^+^ T-lymphocyte count was 320.00 cells/µL (78.50–577.50), and the median CD8^+^ T-lymphocyte count was 500.99 cells/µL (321.00–837.00). Variables with more than 20% missing values—including height, BMI, eGFR, procalcitonin (PCT), TSH, T3, T4, FT3, FT4, phosphorus, and magnesium (Mg²^+^)—were excluded from statistical analysis. Detailed clinical and laboratory data is given in **Table 1**.

**Table 1:** Clinical and laboratory characteristics of the study population.

Variable	No.	Mean ± SD/median (P25, P75)/%
Sex (Male/Female)	117	83/34 (70.9%/29.1%)
Age (years)	117	40.00 (32.00, 54.50)
Body weight (kg)	117	58.11 ± 11.16
Systolic BP (mmHg)	117	119.28 ± 18.32
Diastolic BP (mmHg)	117	77.29 ± 13.49
Duration of HIV diagnosis (months)	117	45.00 (7.50, 87.50)
Hypertension (Yes/No)	117	9/108 (7.7%/92.3%)
Diabetes (Yes/No)	117	7/110 (6.0%/94.0%)
Cortisol (Low/Normal/High)	117	44/61/12 (37.6%/52.1%/10.2%)
ART (Yes/No)	117	98/19 (83.8%/16.2%)
HIV-RNA Viral Load (Below/Above Detection Limit)	117	84/33 (71.8%/28.2%)
Blood cortisol (nmol/L)	117	258.00 (21.75, 431.40)
ACTH (pmol/L)	96	6.33 (2.98, 12.48)
CD4^+^ T lymphocyte count (cells/µL)	117	320.00 (78.50, 577.50)
CD8^+^ T lymphocyte count (cells/µL)	117	500.99 (321.00, 837.00)
White blood cell count (WBC, ×10^9^/L)	117	6.43 ± 2.85
Absolute lymphocyte count (Lymph, ×10^9^/L)	117	1.46 ± 0.82
Lymphocyte % (Lymp%)	117	24.21 ± 11.23
Eosinophil count (Eo, ×10^9^/L)	117	0.08 (0.04, 0.16)
Eosinophil % (Eo%)	117	1.60 (0.70, 2.85)
Hemoglobin (HGB, g/L)	117	118.51 ± 32.06
Red blood cell count (RBC, ×10¹²/L)	117	3.80 ± 0.98
Platelet count (PLT, ×10^9^/L)	117	216.79 ± 101.74
Potassium (K^+^, mmol/L)	117	3.72 ± 0.58
Sodium (Na^+^, mmol/L)	117	137.54 ± 5.04
Calcium (Ca²^+^, mmol/L)	103	2.08 ± 0.37
Random blood glucose (Glu, mmol/L)	117	5.36 ± 1.95
Total bilirubin (TB, µmol/L)	117	11.50 ± 9.80
Direct bilirubin (DB, µmol/L)	117	4.12 (2.34, 5.07)
ALT (U/L)	117	21.00 (14.30, 38.80)
AST (U/L)	117	23.80 (17.40, 34.50)
Triglycerides (TG, mmol/L)	117	1.67 (1.09, 2.27)
Total cholesterol (TC, mmol/L)	117	4.32 ± 1.66
Urea (mmol/L)	117	4.74 (3.60, 6.55)
Creatinine (Cr, µmol/L)	117	84.45 (54.65, 97.50)
HDL-C (mmol/L)	117	1.03 ± 0.43
LDL-C (mmol/L)	117	2.56 ± 1.20

### 3.2 Group comparisons

#### 3.2.1 Comparison of clinical data among groups A, B, and C.

Subjects were stratified into morning serum hypocortisolemia group (Group A), morning serum normocortisolemia (Group B), and abnormal morning serum hypercortisolemia (Group C).

There were statistically significant differences among Groups A, B, and C in diastolic blood pressure, duration of HIV diagnosis, and ART usage rate (P < 0.05).

Diastolic blood pressure was significantly higher in Group A compared with Group B (P = 0.029). The duration of HIV diagnosis was significantly longer in Group A than in both Group B (P = 0.001) and Group C (P = 0.017). The ART usage rate was significantly higher in Group A compared with Group B after adjustment for multiple comparisons (adjusted P = 0.030). No statistically significant differences were observed in pairwise comparisons for the other parameters (adjusted P > 0.05).

There were no significant differences among the three groups in terms of sex, age, body weight, systolic blood pressure, proportion with hypertension, proportion with diabetes, or proportion with HIV-RNA viral load above the detection limit (P > 0.05). Detailed data are presented in **Table 2**.

**Table 2:** Comparison of clinical characteristics among groups A, B, and C.

Variable	Group A (n=44) mean ± SD/median (P25, P75)/n (%)	Group B (n=61) mean ± SD/median (P25, P75)/n (%)	Group C (n=12) mean ± SD/median (P25, P75)/n (%)	F/H/χ²	P
Sex				χ²=1.562	0.458
Male	29 (65.9%)	44 (72.1%)	10 (83.3%)		
Female	15 (34.1%)	17 (27.9%)	2 (16.7%)		
Age (years)	41.93 ± 13.82	46.89 ± 15.53	46.67 ± 18.26	F=1.437	0.242
Body weight (kg)	60.08 ± 10.48	56.91 ± 10.48	56.96 ± 12.89	F=1.102	0.336
Systolic BP (mmHg)	124.48 ± 20.73	116.59 ± 16.22	113.92 ± 15.61	F=3.047	0.051
Diastolic BP (mmHg)	82.18 ± 15.94	73.34 ± 10.41	79.42 ± 12.18	F=6.150	0.003*
Duration of HIV diagnosis (months)	80.50 (36.25, 107.75)	37.00 (2.00, 68.00)	20.50 (1.75, 61.00)	H=15.191	<0.001*
Hypertension (Yes/No)	4 (9.1%)/40 (90.9%)	4 (6.6%)/57 (93.4%)	1 (8.3%)/11 (91.7%)	χ²=0.544	0.885
Diabetes (Yes/No)	3 (6.8%)/41 (93.2%)	3 (4.9%)/58 (95.1%)	1 (8.3%)/11 (91.7%)	χ²=0.782	0.746
ART (Yes/No)	42 (95.5%)/2 (4.5%)	47 (77.0%)/14 (23.0%)	9 (75.0%)/3 (25.0%)	χ²=8.319	0.016*
HIV-RNA above detection limit (Yes/No)	7 (15.9%)/37 (84.1%)	22 (36.1%)/39 (63.9%)	4 (33.3%)/8 (66.7%)	χ²=5.605	0.061

Group A represents PLWH with decreased cortisol.

Group B represents PLWH with normal cortisol levels, and Group C represents PLWH with elevated cortisol levels. *Significant.

#### 3.2.2 Comparison of laboratory test results among groups A, B, and C

There were statistically significant differences among the three groups in ACTH, CD4^+^ T lymphocyte count, WBC, absolute lymphocyte count, eosinophil percentage, triglycerides (TG), urea, and LDL-C (P < 0.05).

Significant differences were observed in several parameters among the study groups. ACTH levels were significantly lower in Group A when compared with Group B (P = 0.026), while CD4^+^ T lymphocyte counts in Group A were significantly lower than in Group C (P = 0.033). Group A also showed significantly higher WBC counts than Group B (P < 0.001), and higher absolute lymphocyte counts when compared with Group B (P = 0.016) and Group C (P = 0.010). The triglyceride levels were significantly higher in Group C than Group B (P = 0.032), whereas urea levels were significantly elevated in Group A compared with Group B (P = 0.020). LDL-C levels were also significantly higher in Group A than in Group C (P = 0.009). No significant differences were found in multiple comparisons of eosinophil percentage among the three groups (P > 0.05), and the remaining pairwise comparisons were also not statistically significant (P > 0.05).

No statistically significant differences were observed among the three groups in CD8^+^ T lymphocyte count, lymphocyte percentage, absolute eosinophil count, hemoglobin (HGB), RBC, PLT, K^+^, Na^+^, Ca²^+^, glucose (Glu), total bilirubin (TB), direct bilirubin (DB), ALT, AST, total cholesterol (TC), creatinine (Cr), or HDL-C (P > 0.05). Detailed data are presented in **Table 3**.

**Table 3:** Comparison of laboratory test results among groups A, B, and C.

Factor	Group A(*n* = 44) $\bar{X}$ *± S/M (P25, P75)*	Group B(*n* = 61) $\bar{X}$ *± S/M (P25, P75)*	Group C(*n* = 12) $\bar{X}$ *± S/M (P25, P75)*	*F/H*	*P*
ACTH (pmol/L)	1.25(0.08, 13.30)	6.58(4.54, 12.16)	10.87(2.91, 14.97)	7.992	0.018
CD4^+^ T lymphocyte count (cells/µL)	422.00(176.25, 601.75)	286.00(78.00, 588.00)	83.50 (40.25, 286.00)	6.878	0.032
CD8+T lymphocyte count (cells/µL)	625.50(411.25, 855.00)	531.00(311.50, 820.00)	435.00(264.00, 1148.00)	2.404	0.301
WBC (10^9^/L)	7.71 ± 2.98	5.58 ± 2.53	6.10 ± 2.27	8.114	**<0.001**
Lymph (10^9^/L)	1.77 ± 0.91	1.33 ± 0.69	1.01 ± 0.71	6.328	0.002
Lymp(%)	24.15(14.30, 34.25)	27.60(18.75, 31.75)	15.25(10.25, 24.25)	4.675	0.097
Eo (10^9^/L)	0.09(0.02, 0.19)	0.08(0.05, 0.17)	0.04(0.02, 0.12)	2.310	0.315
Eo(%)	1.45(0.35, 2.150)	2.00(0.95, 3.40)	0.75(0.33, 3.08)	6.727	**0.035**
HGB (g/L)	125.61 ± 29.67	115.72 ± 33.79	106.67 ± 27.77	2.173	0.119
RBC (1012/L)	3.98 ± 0.93	3.76 ± 1.00	3.37 ± 1.00	1.987	0.142
PLT (1012/L)	216.36 ± 92.14	222.23 ± 110.99	190.75 ± 88.71	0.476	0.622
K+ (mmol/L)	3.78 ± 0.50	3.72 ± 0.56	3.49 ± 0.89	1.166	0.315
Na+ (mmol/L)	136.88 ± 6.13	138.12 ± 4.29	136.95 ± 4.00	0.859	0.426
Ca2+(mmol/L)	2.16 ± 0.25	2.05 ± 0.41	1.92 ± 0.42	2.343	0.101
Glu (mmol/L)	4.99(4.36, 5.63)	4.81(4.45, 5.23)	4.62(4.26, 6.38)	0.284	0.868
TB (µmol/L)	9.99(6.58, 14.25)	8.87(4.99, 15.34)	8.45(6.10, 12.74)	0.504	0.777
DB (µmol/L)	4.12(2.34, 4.95)	4.20(2.16, 5.43)	3.72(2.88, 6.12)	0.120	0.942
ALT (U/L)	22.50(17.48, 47.50)	19.40(14.00, 32.10)	24.00(9.40, 39.45)	3.393	0.183
AST (U/L)	23.30(16.18, 30.80)	23.60(17.65, 39.05)	25.30(14.80, 43.35)	0.429	0.807
TG (mmol/L)	2.28 ± 1.32	1.64 ± 0.93	3.57 ± 6.77	3.624	**0.030**
TC (mmol/L)	4.71 ± 1.31	4.08 ± 1.60	4.11 ± 2.72	1.926	0.150
Urea (mmol/L)	5.26(4.18, 8.25)	4.20(3.50, 5.50)	4.71(3.73, 6.38)	7.425	0.024
Cr (µmol/L)	78.75(58.05, 93.30)	77.50(52.75, 99.20)	82.20 (51.45, 108.18)	0.329	0.848
HDL-C (mmol/L)	1.11 ± 0.44	1.02 ± 0.43	0.79 ± 0.29	2.873	0.061
LDL-C (mmol/L)	2.86 ± 1.06	2.51 ± 1.29	1.71 ± 0.80	4.684	**0.011**

Group A represents PLWH with decreased cortisol, Group B represents PLWH with normal cortisol, and Group C represents PLWH with elevated cortisol.

Bold values shows significant.

### 3.3 Multivariate logistic regression analysis of factors associated with cortisol abnormalities

Cortisol status (low or decreased, normal, elevated) was set as the dependent variable (decreased = 0, normal = 1, elevated = 2). Based on the results of group comparisons, the following variables were included as independent variables: duration of HIV diagnosis, systolic blood pressure, diastolic blood pressure, ART use, ACTH, whether HIV-RNA viral load exceeded the detection limit, CD4^+^ T lymphocyte count, WBC, absolute lymphocyte count, eosinophil percentage, urea, triglycerides (TG), HDL-C, and LDL-C.

An ordered multivariate logistic regression analysis was performed. The resulting model was statistically significant (likelihood ratio test: χ² = 138.00, P = 0.008). The proportional odds assumption was assessed using the test of parallel lines. The result was not statistically significant (χ² = 18.998, P = 0.123), indicating that the proportional odds assumption was not violated and supporting the ordinal logistic regression model.

In the comparison between the decreased cortisol group and the normal/elevated cortisol groups, the intercept β = -5.95 (SE = 2.12, P = 0.005), indicating a significant distinction between the decreased group and the other groups after controlling for other variables. In the comparison between the normal and elevated cortisol groups, the intercept β = -1.99 (SE = 2.02, P = 0.325), indicating no statistically significant difference and suggesting that the boundary between the normal and elevated cortisol groups is less clear.

Among the 14 variables included in the model, duration of HIV diagnosis was significantly associated with cortisol category (OR = 0.987, 95% CI: 0.97–0.99, P = 0.042), indicating that a longer disease duration acts as a suppressive factor for the progression of cortisol from decreased → normal and normal → elevated (**Table 4**).

**Table 4:** Ordered multivariate logistic regression analysis of factors associated with cortisol abnormalities.

Parameter	β^a^	SE	95% CI (OR)	Wald	df	P value^b^
Threshold parameters						
Decreased cortisol vs. Normal/Elevated	-5.83	2.12	(-9.99, -1.67)	7.56	1	0.006*
Normal cortisol vs. Elevated	-1.85	2.03	(-5.84, 2.13)	0.83	1	0.361
Location parameters						
Duration of HIV diagnosis (months)	-0.014	0.007	(0.97–0.99)	3.88	1	0.049*

^a^β represents the log odds ratio. The odds ratio (OR) can be obtained by exponentiating β (for example, for duration of HIV diagnosis, OR = 0.987). *Significant.

^b^P < 0.05 indicates statistical significance.

Bold values shows significant.

### 3.4 Phase II: results

#### 3.4.1 Clinical data and laboratory results

Among the ART groups, 22 PLWH (30.1%) received regimen 1, 22 PLWH (30.1%) received regimen 2, and 29 PLWH (39.8%) received regimen 3. The median age of the study population was 38.00 years (interquartile range, 31.50–49.00), and the median blood cortisol level was 133.00 nmol/L (17.07–325.90). The median ACTH level was 5.33 pmol/L (1.11–11.86), and the median CD4+ T lymphocyte count was 411.00 cells/µL (171.50–618.50). Detailed information is presented in **Table 5**.

**Table 5:** Clinical data and laboratory results of the study population.

Parameter	No.	Mean ± SD/Median (P25, P75)/n (%)*
Gender, Male/Female	73	49/24 (67.1%/32.9%)
Age (years)	73	38.00 (31.50, 49.00)
Systolic BP (mmHg)	73	117.16 ± 15.73
Diastolic BP (mmHg)	73	77.45 ± 14.64
Weight (kg)	73	58.57 ± 11.35
Duration since HIV/AIDS diagnosis (months)	73	56.50 (18.00, 86.75)
Hypertension, Yes/No	73	4/69 (5.5%/94.5%)
Diabetes, Yes/No	73	3/70 (4.1%/95.9%)
ART regimen (1)/(2)/(3)	73	22/22/29 (30.1%/30.1%/39.8%)
Blood cortisol, Low	73	35 (47.9%)
Blood cortisol, Normal	73	38 (52.1%)
Blood cortisol (nmol/L)	73	133.00 (17.07, 325.90)
ACTH (pmol/L)	62	5.33 (1.11, 11.86)
HIV-RNA above detection limit	73	14 (19.2%)
CD4+ T lymphocyte count (cells/µL)	73	411.00 (171.50, 618.50)
CD8+ T lymphocyte count (cells/µL)	73	551.00 (335.50, 835.00)
WBC (10^9^/L)	73	6.52 ± 2.80
Lymphocytes (10^9^/L)	73	1.39 (1.02, 2.09)
Lymphocytes (%)	73	27.60 (17.25, 34.35)
Eosinophils (10^9^/L)	73	0.07 (0.04, 0.13)
Eosinophils (%)	73	1.50 (0.65, 2.40)
Hemoglobin (g/L)	73	124.78 ± 30.85
RBC (10¹²/L)	73	3.93 ± 0.94
Platelets (10¹²/L)	73	216.00 (161.50, 272.00)
K+ (mmol/L)	73	3.80 ± 0.55
Na+ (mmol/L)	73	137.30 ± 5.01
Glucose (mmol/L)	73	5.38 ± 1.99
Total bilirubin (µmol/L)	73	9.98 (6.35, 14.12)
Direct bilirubin (µmol/L)	73	4.22 (2.46, 4.93)
ALT (U/L)	73	21.90 (15.90, 39.40)
AST (U/L)	73	22.90 (17.00, 30.70)
Triglycerides (TG, mmol/L)	73	1.65 (1.05, 2.34)
Total cholesterol (TC, mmol/L)	73	4.33 (3.70, 5.50)
Creatinine (Cr, µmol/L)	73	77.00 (52.05, 92.65)
Urea (mmol/L)	73	4.74 (3.60, 6.33)
HDL-C (mmol/L)	73	1.13 ± 0.40
LDL-C (mmol/L)	73	2.79 ± 1.13

*Values below detection limit calculated as 0. ART regimen Group (1): Nucleoside Reverse Transcriptase Inhibitors + Non-Nucleoside Reverse Transcriptase Inhibitors (NRTIs + NNRTIs). ART regimen Group (2): Nucleoside Reverse Transcriptase Inhibitors + Protease Inhibitors (NRTIs + PIs). ART regimen Group (3): Nucleoside Reverse Transcriptase Inhibitors + Integrase Strand Transfer Inhibitors (NRTIs + INSTIs). *Some values were below the detection limit and were calculated as 0 for the median.

#### 3.4.2 Comparisons between groups

##### 3.4.2.1 Comparison of clinical data among different ART regimen groups

Significant differences were observed in age (H = 6.679, P < 0.05) and the proportion of PLWH with decreased cortisol (H = 6.679, P < 0.05) among the different ART regimen groups. Specifically, the age in Group (2) (NRTIs + PIs) was significantly higher than in Group (3) (NRTIs + INSTIs) (adjusted P = 0.030). The proportion of PLWH with decreased cortisol in Group (2) was significantly higher than in Group (1) (NRTIs + NNRTIs) (adjusted P = 0.018) and Group (3) (adjusted P = 0.006). No significant differences were observed for the other pairwise comparisons (adjusted P > 0.05).

For other clinical variables, including sex, body weight, duration since HIV diagnosis, systolic blood pressure, diastolic blood pressure, prevalence of hypertension or diabetes, and proportion with HIV-RNA viral load above the detection limit, no statistically significant differences were found among the groups (P > 0.05). Detailed data are shown in **Table 6**; **Supplementary Table 1**.

**Table 6:** Comparison of clinical data among different ART regimen groups.

Variable	Group (1) (NRTIs + NNRTIs, n=22)	Group (2) (NRTIs + PIs, n=22)	Group (3) (NRTIs + INSTIs, n=29)	H/F/χ²	P value
Sex, male/female	14/8 (63.6%/36.4%)	15/7 (68.2%/31.8%)	20/9 (69.0%/31.0%)	0.112	0.945
Age (years)	35.50 (30.00, 45.50)	43.00 (35.50, 52.00)	37.00 (30.50, 48.50)	6.679	<0.05*
Weight (kg)	57.80 ± 10.25	59.10 ± 11.32	59.00 ± 12.10	0.245	0.783
Duration of HIV/AIDS (months)	55.00 (20.00, 85.00)	60.00 (15.50, 87.00)	53.50 (18.00, 88.00)	0.455	0.796
Systolic BP (mmHg)	116.50 ± 15.30	118.90 ± 16.02	116.90 ± 15.80	0.347	0.708
Diastolic BP (mmHg)	76.80 ± 13.80	78.50 ± 15.20	77.00 ± 14.30	0.122	0.885
Hypertension, yes/no	1/21 (4.5%/95.5%)	2/20 (9.1%/90.9%)	1/28 (3.4%/96.6%)	0.562	0.754
Diabetes, yes/no	1/21 (4.5%/95.5%)	1/21 (4.5%/95.5%)	1/28 (3.4%/96.6%)	0.038	0.981
Cortisol decreased, yes/no	7/15 (31.8%/68.2%)	14/8 (63.6%/36.4%)	14/15 (48.3%/51.7%)	6.679	<0.05*
HIV-RNA above detection limit, yes/no	5/17 (22.7%/77.3%)	5/17 (22.7%/77.3%)	4/25 (13.8%/86.2%)	1.126	0.57

*Significant difference P < 0.05, Cortisol decreased: morning cortisol < 133 nmol/L, Group (1): NRTIs + NNRTIs; Group (2): NRTIs + PIs; Group (3): NRTIs + INSTIs.

##### 3.4.2.2 Comparison of test results among different ART regimen groups

The overall distributions of absolute eosinophil count, eosinophil percentage, RBC, PLT, TB, DB, TG, Cr, and HDL-C differed significantly among the different ART groups (P < 0.05). The absolute eosinophil count and eosinophil percentage were significantly higher in Group (1) than in Group (2) (adjusted P = 0.040 and 0.023, respectively). In contrast, RBC and PLT levels were significantly lower in Group (2) compared with Group (3) (P = 0.001 and adjusted P = 0.017, respectively). Total bilirubin (TB) and direct bilirubin (DB) levels were significantly lower in Group (1) than in Group (2) (adjusted P = 0.012 and 0.020, respectively). Additionally, triglyceride (TG) levels were significantly higher in Group (2) than in Group (3) (adjusted P = 0.017). Creatinine (Cr) and HDL-C levels were significantly lower in Group (1) compared with Group (3) and Group (2), respectively (adjusted P = 0.032 and 0.046).No statistically significant differences were observed for the remaining comparisons among groups (P > 0.05). For blood cortisol, ACTH, CD4^+^ T lymphocyte count, CD8^+^ T lymphocyte count, WBC, absolute lymphocyte count, lymphocyte percentage, HGB, K^+^, Na^+^, Glu, AST, ALT, TC, Urea, and LDL-C, no significant differences were found among the groups (P > 0.05). See **Table 7** for details.

**Table 7:** Comparison of test results among different ART regimen groups.

Parameter	Group (1) (n=22) $\bar{X}$ ± S/M (P25, P75)	Group (2) (n=22) $\bar{X}$ ± S/M (P25, P75)	Group (3) (n=29) $\bar{X}$ ± S/M (P25, P75)	F/H	P
Blood cortisol (nmol/L)	256.65 (19.89, 370.55)	95.98 (7.47, 132.31)	229.20 (17.41, 338.75)	3.889	0.143
ACTH (pmol/L)	7.39 (3.06, 16.63)	2.13 (0, 9.62)	6.39 (2.13, 10.87)	5.449	0.066
CD4^+^ T lymphocytes (cells/µL)	296.00 (120.00, 638.25)	420.50 (178.75, 521.50)	535.00 (146.00, 672.00)	1.125	0.57
CD8^+^ T lymphocytes (cells/µL)	501.00 (357.00, 742.50)	605.50 (437.25, 980.50)	522.00 (249.00, 878.00)	1.807	0.405
WBC (10^9^/L)	6.35 ± 3.39	6.46 ± 2.70	6.69 ± 2.47	0.098	0.906
Lymphocytes (10^9^/L)	1.31 (1.06, 1.70)	1.38 (1.02, 1.84)	1.88 (0.84, 2.23)	2.186	0.335
Lymphocytes (%)	24.15 (17.38, 31.25)	25.05 (14.43, 35.08)	28.90 (18.90, 35.80)	1.029	0.598
Eosinophils (10^9^/L)	0.10 (0.07, 0.16)	0.05 (0.03, 0.10)	0.08 (0.05, 0.14)	6.323	**0.042**
Eosinophils (%)	1.80 (1.33, 3.03)	0.65 (0.38, 1.75)	1.50 (0.85, 2.80)	7.313	**0.026**
HGB (g/L)	119.86 ± 33.89	118.64 ± 23.33	133.17 ± 32.48	1.831	0.168
RBC (10¹²/L)	3.82 ± 0.92	3.46 ± 0.75	4.37 ± 0.92	7.033	**0.002**
PLT (10¹²/L)	218.00 (158.25, 246.00)	180.50 (120.00, 241.75)	245.00 (196.00, 283.00)	7.678	**0.022**
K^+^ (mmol/L)	3.92 ± 0.54	3.64 ± 0.61	3.83 ± 0.51	1.53	0.224
Na^+^ (mmol/L)	137.71 ± 4.12	135.56 ± 6.86	138.31 ± 3.62	2.047	0.137
Glucose (Glu, mmol/L)	5.15 ± 2.29	5.55 ± 2.26	5.42 ± 1.53	0.227	0.797
TB (µmol/L)	6.35 (3.78, 10.82)	11.77 (8.67, 16.61)	10.46 (6.80, 13.70)	8.408	**0.015**
DB (µmol/L)	3.13 (1.71, 4.74)	4.30 (4.05, 7.28)	4.22 (2.59, 4.92)	7.492	**0.024**
ALT (U/L)	19.30 (13.85, 39.65)	20.35 (15.10, 37.50)	23.70 (18.45, 46.85)	2.173	0.337
AST (U/L)	22.35 (17.15, 30.38)	21.90 (16.05, 27.50)	23.00 (18.30, 39.15)	1.248	0.536
TG (mmol/L)	1.59 (1.02, 2.03)	2.24 (1.51, 3.10)	1.49 (0.99, 1.79)	7.675	**0.022**
TC (mmol/L)	4.02 (3.54, 5.06)	5.37 (3.97, 6.16)	4.33 (3.88, 5.10)	5.72	0.057
Urea (mmol/L)	4.55 (3.70, 5.68)	4.82 (4.13, 7.51)	4.20 (3.45, 6.25)	1.697	0.428
Cr (µmol/L)	59.90 (48.20, 81.10)	60.20 (44.13, 95.15)	85.20 (77.00, 99.95)	8.204	**0.017**
HDL-C (mmol/L)	1.02 ± 0.46	1.31 ± 0.40	1.09 ± 0.31	3.418	**0.038**
LDL-C (mmol/L)	2.46 ± 0.95	3.01 ± 1.29	2.89 ± 1.11	1.46	0.239

Group (1) regimen: NRTIs + NNRTIs, Group (2) regimen: NRTIs + PIs, Group (3) regimen: NRTIs + INSTIs.

Bold values shows significant.

### 3.5 Forward: LR binary logistic regression analysis of factors associated with reduced blood cortisol

Blood cortisol reduction was taken as the dependent variable (normal = 0, reduced = 1). Based on the univariate analysis, the following variables were selected as independent variables: sex, different ART regimens, age, duration of HIV diagnosis, ACTH, RBC, PLT, absolute eosinophil count, eosinophil percentage, TB, DB, Cr, TG, and HDL-C. A forward: LR (likelihood ratio) binary logistic regression analysis was performed.

The final logistic regression model was statistically significant (likelihood ratio test: χ² = 16.63, P = 0.001). Among the 14 variables included in the model; The longer the duration of HIV diagnosis, the higher the likelihood of cortisol reduction, with statistical significance (OR = 1.02, 95% CI: 1.00–1.03, P = 0.021). Compared with regimen (1), the use of regimen (2) significantly increased the risk of cortisol reduction. It was identified as an independent risk factor for reduced cortisol, with statistical significance (OR = 5.36, 95% CI: 1.13–25.34, *P* = 0.034). See **Table 8** for details.

**Table 8:** Forward: LR binary logistic regression analysis of factors associated with reduced blood cortisol.

Variable	β	SE	Wald	P value	OR	95% CI
Duration of HIV diagnosis (months)	0.02	0.007	5.32	**0.021**	1.02	1.00–1.03
ART regimen ①	–	–	7.36	**0.025**	–	–
ART regimen ②	1.68	0.79	4.49	**0.034**	5.36	1.13–25.34
ART regimen ③	0.3	0.69	0.19	0.661	0.74	0.19–2.85
Constant	–1.63	0.67	5.84	**0.016**	0.19	–

Group ① regimen: NRTIs + NNRTIs, Group ② regimen: NRTIs + PIs, Group ③ regimen: NRTIs + INSTIs. Group (1) was set as the reference category, a) β = log odds ratio, b) This table only includes statistically significant variables, c) P < 0.05 indicates statistical significance.

Bold values shows significant.

## 4 Discussion

This study explored the factors associated with abnormal serum cortisol levels among PLWH and evaluated the relationship between different ART regimens and cortisol alterations. The PLWH in the cortisol-lowered group had a significantly longer duration of HIV/AIDS compared with the normal and elevated cortisol groups. Multivariate regression analysis confirmed a negative association between disease duration and cortisol levels, suggesting that prolonged HIV infection may impair adrenal function via multiple mechanisms. Previous studies have also reported elevated basal cortisol levels in many PLWH, often attributed to chronic inflammation and glucocorticoid resistance, characterized by HPA axis hyperactivity and increased ACTH secretion (25, 26). Mayo et al. reported that adrenal insufficiency is predominantly observed in advanced stages of HIV infection, despite frequent subclinical HPA-axis abnormalities throughout the course of the disease (27). Likewise, a retrospective study of AIDS patients by Fernández-Ruiz et al. found adrenal insufficiency in 22% of PLWH, highlighting the association between prolonged and advanced PLWH disease (28). More recently, a prospective cohort study reported a high prevalence of adrenal insufficiency among PLWH, identified hormonal abnormalities, including altered cortisol dynamics, and concluded that adrenal dysfunction remains an underrecognized complication requiring active screening in this population (29). In addition, studies evaluating long-term glucocorticoid exposure in PLWH have shown that cortisol regulation is closely linked to HIV disease progression and immune status (30).

Chronic psychological and physiological stress associated with early HIV infection, including the impact of disease, social stigma, economic burden, and mental health challenges, may also activate the HPA axis and elevate cortisol secretion (24, 31, 32). Repeated cortisol surges and chronic HPA activation may eventually result in adrenal hypofunction (25, 26, 33, 34).

PLWH with elevated cortisol showed higher ACTH levels and shorter HIV duration, potentially reflecting early HPA axis activation during initial HIV infection (20, 26, 35). Opportunistic infections involving the hypothalamus, pituitary, or adrenal glands such as cytomegalovirus, *Mycobacterium tuberculosis*, Cryptococcus, *Toxoplasma gondii*, *Pneumocystis jirovecii*, and SARS-CoV-2, may also cause adrenal structural damage, leading to decreased cortisol levels (20). However, widespread ART has greatly reduced the incidence of direct adrenal infections. Although cumulative damage from past infections cannot be entirely excluded, it likely plays a minor role in the current cohort (36, 37).

Given that low cortisol often presents with nonspecific clinical manifestations and delayed diagnosis may result in severe complications and death, we recommend routine adrenal function monitoring in PLWH with long-standing HIV/AIDS, particularly those with a disease duration exceeding 7 years. Morning cortisol and ACTH levels should be measured every 6–12 months, and an ACTH stimulation test should be considered when indicated to evaluate adrenal reserve.

Cortisol-lowered PLWH also exhibited higher diastolic blood pressure and longer HIV duration compared with normal cortisol PLWH, which may be related to cumulative cardiovascular and neuroendocrine damage from chronic inflammation.

Metabolic changes provide additional insight into cortisol dysregulation. PLWH with elevated cortisol exhibited significantly higher triglyceride levels, consistent with glucocorticoid-induced metabolic syndrome (38–40). Glucocorticoids enhance lipolysis and hepatic triglyceride synthesis via increased gluconeogenesis and free fatty acid mobilization (39, 41). Conversely, cortisol-lowered PLWH had higher urea and LDL-C levels, potentially due to enhanced protein catabolism and impaired hepatic LDL receptor-mediated uptake in low-cortisol states. These findings indicate that PLWH with cortisol dysregulation may be at increased risk of renal injury (elevated urea), atherosclerosis (elevated LDL-C), and fatty liver (triglyceride accumulation), suggesting the need for personalized interventions.

Our findings suggest a possible association between ART exposure and altered cortisol homeostasis; however, the mechanisms remain uncertain. Protease inhibitors, ritonavir-containing regimens, have been reported to affect cytochrome P450-mediated steroid metabolism and represent a biologically plausible explanation for altered cortisol concentrations. However, cortisol regulation is multifactorial; the lower cortisol levels observed in some ART-treated participants cannot be attributed solely to ART (42–45). In this study, cortisol-lowered PLWH had the longest HIV duration, the highest proportion receiving ART, higher CD4^+^ T-cell counts, and the lowest proportion with detectable viral loads. These findings may reflect (1): ART-mediated viral suppression reducing inflammatory burden, which alters cytokine secretion patterns (e.g., IL-6, TNF-α) and indirectly influences HPA axis regulation; and (2) irreversible adrenal damage from chronic infection despite viral control, consistent with findings from a cross-sectional study in Nigeria (46).

Currently, there are seven major classes of more than 40 antiretroviral drugs internationally, including nucleoside/nucleotide reverse transcriptase inhibitors (NRTIs), non-nucleoside reverse transcriptase inhibitors (NNRTIs), protease inhibitors (PIs), integrase strand transfer inhibitors (INSTIs), fusion inhibitors (FIs), C-C chemokine receptor type 5 inhibitors (CCR5Is), and capsid inhibitors [8]. In China, the main antiretroviral drug classes include NRTIs, NNRTIs, PIs, INSTIs, and FIs (including combination formulations).

In our hospital, the commonly used regimen includes two or one NRTI backbone drugs, mainly lamivudine (3TC) + tenofovir disoproxil fumarate (TDF), with a small proportion of PLWH using abacavir (ABC) or zidovudine (AZT). The third drug is one of the following: 1) Efavirenz (EFV, NNRTI), 2) Lopinavir/ritonavir (LPV/r, PI), and 3) Dolutegravir (DTG, INSTI) Biktarvy, the currently available smallest single-tablet regimen based on INSTI, contains bictegravir (INSTI) + emtricitabine/tenofovir alafenamide (NRTIs), belonging to group (3). One patient in our cohort used a regimen of three NRTIs; however, because this group had only one patient, statistical power was insufficient, and this group was excluded from the second part of the study analysis.

Although ART regimen (2) (NRTIs + PIs) was identified as an independent factor associated with reduced cortisol levels, however, these findings should be interpreted cautiously. Other factors, including acute illness, physiological stress, inflammatory status, concomitant medications, liver function, and other comorbid conditions, influence serum cortisol levels (47, 48). Although major endocrine disorders and glucocorticoid exposures were excluded, residual confounding cannot be excluded in this retrospective cross-sectional study. Therefore, the observed association between ART and cortisol levels does not establish causality and may partly reflect differences in disease severity, duration of HIV infection, or other unmeasured clinical factors.

Interestingly, reduced cortisol levels had longer HIV duration, higher rates of ART use, higher CD4^+^ T-cell counts, and a lower proportion of detectable viral loads (30). These characteristics are more consistent with successful viral suppression than with acute systemic illness. However, because markers of acute stress, inflammatory cytokines, and detailed comorbidity data were not available, we cannot exclude the possibility that cortisol alterations were influenced by underlying clinical status rather than the ART regimen alone. Future studies incorporating inflammatory markers, cortisol-binding globulin measurements, and longitudinal endocrine assessments will be necessary to clarify whether the observed associations reflect ART-related effects.

In recent years, there have been case reports of iatrogenic Cushing’s syndrome or an association with serum cortisol categories in PLWH co-administered PIs with exogenous glucocorticoids (42–45, 49). Cohort studies reported that approximately 5% of PLWH receiving intra-articular steroids during PI therapy (especially ritonavir) developed persistent HPA axis suppression (50). The mechanism is that PIs are potent CYP3A4 inhibitors, which significantly reduce the clearance of corticosteroids metabolized by this enzyme (e.g., fluticasone, triamcinolone), resulting in increased blood concentrations and prolonged half-life (51, 52). Long-term exposure to high corticosteroid levels suppresses the HPA axis via negative feedback, reducing endogenous cortisol secretion and, eventually, leading to adrenal atrophy and biochemical cortisol abnormalities. A meta-analysis found that approximately 4.2% of nasal and 7.8% of inhaled corticosteroid users among PLWH developed HPA axis suppression (53). However, review of patient records in this study revealed that PLWH using PIs did not have documented exogenous corticosteroid use. Possible reasons include: a) PLWH used exogenous corticosteroids but did not report them to physicians; b) PIs themselves may cause cortisol metabolism abnormalities; c) PLWH on regimen (2) had the longest duration of HIV/AIDS, which, although not statistically significant, might increase the risk of cortisol reduction.

Regardless of the cause, clinicians should remain vigilant for biochemical cortisol abnormalities in PLWH receiving PIs. In this study, only 22 PLWH used PIs, and the small sample size and few normal cortisol cases limited statistical power, preventing further multivariate analysis of factors related to cortisol reduction in PI users. Future large-scale prospective or cohort studies are needed to further explore the influencing factors.

The findings from Phase II subgroup analysis derived from the Phase I cohort, provide insight into the potential relationship between ART regimens and cortisol abnormalities in PLWH. Phase II specifically evaluated PLWH receiving stable ART regimens to further explore whether treatment patterns were associated with reduced cortisol levels. Compared with NRTIs + NNRTIs, the regimen consisting of NRTIs + PIs was associated with a significantly higher proportion of decreased cortisol and remained independently associated with cortisol reduction in multivariable logistic regression. These findings suggest that the composition of the ART regimen may influence HPA axis function and cortisol metabolism. Some of the biological mechanisms may explain these observations. The chronic HIV infection is associated with persistent immune activation, inflammation, and neuroendocrine dysregulation, which may contribute to alterations in cortisol secretion and ACTH responses in PLWH (54). In the present study, lower ACTH levels may show impaired HPA axis regulation and a persistent inflammatory process. Long-term immune activation and chronic inflammation during HIV infection have also been shown to persist despite suppressive ART (55). Moreover, protease inhibitors may interfere with cytochrome P450-mediated steroid metabolism, contributing to cortisol abnormalities (56).

Alterations in cortisol and ACTH levels may reflect dysregulation of the HPA axis in PLWH. Chronic HIV infection is associated with persistent immune activation, systemic inflammation, and neuroendocrine dysfunction, which may contribute to abnormal cortisol secretion and altered ACTH in individuals receiving suppressive ART. In our study, differences in ACTH levels and immune cell distributions among cortisol groups may indicate complex interactions between endocrine regulation, chronic inflammation, and immune status. The protease inhibitor–based regimens may influence HPA axis function through cytochrome P450 enzyme activity, contributing to the higher frequency of decreased cortisol (56).

## 5 Study limitations

1. This was a retrospective study rather than a prospective cohort study, so the causal relationship between ART and abnormal serum cortisol could not be established.
2. Cortisol was measured by immunoassay rather than mass spectrometry. Given the known susceptibility of immunoassays to interference from binding protein variability, particularly alterations in Corticosteroid-Binding Globulin (CBG) in patients with possible liver disease, the cortisol results should be interpreted with caution.
3. Dynamic functional assessments of adrenal reserve, such as ACTH stimulation tests and circadian rhythm evaluations of serum cortisol, were lacking, and urinary or salivary cortisol levels were not measured.
4. Data for some PLWH were incomplete and may contain certain errors.
5. This was a single-center study with a relatively small sample size, which may introduce bias.
6. The relatively small sample size in the current study limits the ability to perform detailed mechanistic analyses of the interactions among hypothalamic–pituitary–adrenal (HPA) axis dysregulation, chronic inflammation, and the potential effects of ART regimens on cortisol abnormalities in PLWH.
7. Multicollinearity among the independent variables included in the regression analyses was not formally evaluated, which may have influenced the stability of some model estimates.
8. The number of participants in the elevated cortisol group was relatively small in both Phase I and Phase II analyses, which may reduce the statistical stability of estimates and limit the reliability of subgroup comparisons. Therefore, results related to the elevated cortisol group should be interpreted with caution. In addition, cortisol was analyzed as a categorical variable; future studies with larger sample sizes may consider treating it as a continuous variable to further validate these findings.

The factors associated with abnormal serum cortisol levels in PLWH, as well as the relationship between different ART regimens and cortisol reduction, remain to be confirmed in larger cohorts and prospective studies.

## 6 Conclusion

This study investigated the distribution of serum cortisol categories and their associations with clinical characteristics and antiretroviral therapy (ART) regimens in people living with HIV (PLWH). Differences in clinical and laboratory parameters were observed across ART regimens; however, the overall effect sizes were modest, and these findings should be interpreted in the context of a retrospective cross-sectional design. More than one-third of PLWH in this cohort exhibited decreased serum cortisol levels. In multivariable analysis, longer duration of HIV infection was independently associated with serum cortisol category. In ART regimens, protease inhibitor–based therapy (NRTIs + PIs) was associated with higher odds of decreased serum cortisol compared with NNRTI- or INSTI-based regimens. Overall, these findings suggest an association between HIV-related clinical characteristics, ART regimens, and serum cortisol levels. Further prospective studies with dynamic endocrine assessment are required to clarify these relationships.

## Data Availability

The original contributions presented in the study are included in the article/**Supplementary Material**. Further inquiries can be directed to the corresponding author.
