# Supplementary material for: Association of antiretroviral therapy regimens with serum cortisol abnormalities in people living with HIV/AIDS: a retrospective study
Source: Front Endocrinol (Lausanne). 2026 Jun 26;17:1841173. doi: 10.3389/fendo.2026.1841173 (PMC13350351; doi:10.3389/fendo.2026.1841173)
Supplement: Supplementary file 1 [file Table1.docx]

**Table S1. Clinical data among different groups of ART regimens.**

| **Variable** | **Group ① (NRTIs + NNRTIs, n=22)** | **Group ② (NRTIs + PIs, n=22)** | **Group ③ (NRTIs + INSTIs, n=29)** | **F/H / χ²** | **P value** |
| --- | --- | --- | --- | --- | --- |
| Cortisol | 10.87 | 0.004* |  |  |  |
| ↓ (Decreased) | 8 (36.4%) | 17 (77.3%) | 10 (34.5%) |  |  |
| Normal | 14 (63.6%) | 5 (22.7%) | 19 (65.5%) |  |  |
| HIV-RNA above detection limit | 0.149 | 0.965 |  |  |  |
| Yes | 4 (18.2%) | 4 (18.2%) | 6 (20.7%) |  |  |
| No | 18 (81.8%) | 18 (81.8%) | 23 (79.3%) |  |  |

**Notes:** Group 1: NRTIs + NNRTIs, Group 2: NRTIs + PIs, Group ③: NRTIs + INSTIs, *Statistically significant, P < 0.05
